# Nasopharyngeal Bacterial Colonization and Gene Polymorphisms of Mannose-Binding Lectin and Toll-Like Receptors 2 and 4 in Infants

**DOI:** 10.1371/journal.pone.0026198

**Published:** 2011-10-13

**Authors:** Juho Vuononvirta, Laura Toivonen, Kirsi Gröndahl-Yli-Hannuksela, Alex-Mikael Barkoff, Laura Lindholm, Jussi Mertsola, Ville Peltola, Qiushui He

**Affiliations:** 1 Department of Infectious Disease Surveillance and Control, National Institute for Health and Welfare, Turku, Finland; 2 Department of Pediatrics, Turku University Hospital, Turku, Finland; 3 Turku Institute for Child and Youth Research, University of Turku, Turku, Finland; 4 Department of Medical Microbiology and Immunology, University of Turku, Turku, Finland; Charité-University Medicine Berlin, Germany

## Abstract

**Background:**

Human nasopharynx is often colonized by potentially pathogenic bacteria. Gene polymorphisms in mannose-binding lectin (MBL), toll-like receptor (TLR) 2 and TLR4 have been reported. The present study aimed to investigate possible association between nasopharyngeal bacterial colonization and gene polymorphisms of MBL, TLR2 and TLR4 in healthy infants.

**Methodology/Principal Findings:**

From August 2008 to June 2010, 489 nasopharyngeal swabs and 412 blood samples were taken from 3-month-old healthy Finnish infants. Semi-quantitative culture was performed and pyrosequencing was used for detection of polymorphisms in MBL structural gene at codons 52, 54, and 57, TLR2 Arg753Gln and TLR4 Asp299Gly. Fifty-nine percent of subjects were culture positive for at least one of the four species: 11% for *Streptococcus pneumoniae*, 23% for *Moraxella catarrhalis*, 1% for *Haemophilus influenzae* and 25% for *Staphylococcus aureus*. Thirty-two percent of subjects had variant types in MBL, 5% had polymorphism of TLR2, and 18% had polymorphism of TLR4. Colonization rates of *S. pneumoniae* and *S. aureus* were significantly higher in infants with variant types of MBL than those with wild type (p = .011 and p = .024). Colonization rates of *S. aureus* and *M. catarrhalis* were significantly higher in infants with polymorphisms of TLR2 and of TLR4 than those without (p = .027 and p = .002).

**Conclusions:**

Our study suggests that there is an association between nasopharyngeal bacterial colonization and genetic variation of MBL, TLR2 and TLR4 in young infants. This finding supports a role for these genetic variations in susceptibility of children to respiratory infections.

## Introduction

Nasopharynx is a complex ecosystem and contains various bacteria. These bacteria can asymptomatically colonize infants and young children but are also associated with respiratory diseases [1], [2]. Bacterial respiratory infections are mainly caused by extracellular encapsulated bacteria like *Streptococcus pneumoniae*, *Haemophilus influenzae*, or *Moraxella catarrhalis*. *S. pneumoniae* and *M. catarrhalis* can colonize up to 54% and 72% of children, respectively, by 1 year of age [3]. *Staphylococcus aureus* colonizes up to 35% of young children and is associated with wide range of diseases [4]. Infants younger than 3 months of age, who have been colonized by these pathogens, have a greater risk in developing respiratory infections in the next 6 to 9 months of their early life [5].

One major family of the host proteins are Toll-like receptors (TLRs), which initiate signaling of innate immune response and link innate and adaptive immune systems. Each TLR recognizes specific pathogen-associated molecular patterns (PAMPs) of different microbes [6]. TLR4 is a transmembrane signaling receptor of lipopolysaccharide (LPS) from Gram-negative bacteria, principally expressed in macrophages and dendritic cells. *Tlr4* gene is located on chromosome 9q32-33 [7]. A polymorphic site Asp299Gly occurs in extracellular domain of TLR4 [8], which results in a conformational change of this domain [9] and an impaired response to LPS.

TLR2 is located on chromosome 4q32 and it recognizes numerous ligands, most notably peptidoglycan (PGN), which is the major component of the cell wall of Gram-positive bacteria including *S. aureus* [10]. The most widely characterized single nucleotide polymorphism (SNP) of TLR2 is an amino acid substitution at position 753 from arginine to glutamine. This change in amino acid causes an impairment of TLR2 signaling in response to some ligands potentially increasing susceptibility to infections [11].

Mannose-binding lectin (MBL) is an important protein of innate immune system. It binds to various microorganisms including Gram-positive and Gram-negative bacteria leading to agglutination of microorganisms and clearance by phagocytes. MBL activates complement via lectin pathway. Polymorphisms in MBL gene are known to influence serum MBL concentration. Heterozygote allelic variants in codons 52 (allele D), 54 (allele B) and 57 (allele C) are all located in exon 1 and result in amino acid substitutions and significant reduction of serum MBL concentration, whereas homozygosity or combination of the minority alleles (O/O) result in almost complete deficiency of serum MBL [12]–[13]. It has been shown that low serum MBL concentration is related to a higher risk of respiratory infections [14].

In this study we wanted to investigate whether there is an association between nasopharyngeal bacterial colonization and gene polymorphisms in MBL, TLR2 and TLR4 in healthy infants.

## Materials and Methods

### Study design and study subjects

The present study population was recruited from Finnish children, who are taking part in the ongoing study called Steps to Children's Healthy Development and Wellbeing (STEPS)-study. The STEPS-study is designed as a prospective, observational, cohort study in which approximately 1,800 children are followed-up from before birth.

Healthy infants who visited the study clinic at the age of 3 months, on monday or tuesday during the period between August 2008 and June 2010, were included in the present study. Three-month visit was preferably scheduled before the universal vaccinations at 3 months, resulting in mean age of 0.22 years (standard deviation, 0.08 years). Nasopharyngeal samples (NP) were collected from 489 children by using flocked nasopharyngeal swabs (Copan, Brescia, Italy). After taken, the swab was immersed in 1 mL of 0.9% NaCl, and homogenized by vortexing. The swab was removed from the transport tube, and the tube containing bacterial solution was transported within 3 hours to laboratory for bacterial culture. Blood samples were collected from a total of 412 children.

The study protocol was approved by the Ethics Committee of the Hospital District of the South-Western Finland, Turku, Finland. All participants or parents of participating children gave their written informed consent.

### Bacterial culture

At the laboratory, 10 µl-loopful of bacterial suspension was plated and then spread over one-quarter of the plate, and the sample was streaked onto remaining three quadrants by using the same 10 µl loop. Four different culture plates were used: a blood agar plate containing 5% sheep blood, a heated blood agar (chocolate agar) plate, a *H. influenzae* selective plate (a heated blood agar plate containing 300 mg/l bacitracin) and a *S. pneumoniae* selective plate (sheep blood agar plate containing 5 mg/l colistin and 2,5 mg/l oxolinic acid). Plates were incubated in 5% CO_2_ at 35°C for 48 hours. Plates were examined daily for the growth of different bacterial species. Suspected colonies of each species were identified as follows: *S. pneumoniae* isolates by using the optochin disk susceptibility test (Oxoid, Basingstoke, England), *H. influenzae* isolates by the X, V and X+V factor test (Oxoid), *M. catarrhalis* isolates by the oxidase and Tributyrin test (Rosco Diagnostica, Taastrup, Denmark) and *S. aureus* isolates by the coagulase, catalase and latex agglutination test (Staphaurex, Remel Inc., Lenexa, KS, USA). Other genera or species of bacteria were identified by standard microbiological methods.

### DNA preparation

DNA was extracted from 200 µl of whole blood by QIAGEN QIAamp DNA Blood Mini Kit 250 (Qiagen, Hilden, Germany) according to the manufacturer's protocol.

### Genotyping

The genotyping of MBL, TLR2 and TLR4 was performed by pyrosequencing (PSQ™96MA Pyrosequencer, Biotage, Uppsala, Sweden), using a PSQ™96 Pyro Gold Q96 reagent kit according to the manufacturer's protocol. The PCR and sequencing primers used for the TLR2 Arg753Gln (rs5743708), TLR4 Asp299Gly (rs4986790) and MBL2 gene in codons 52 (allele D, rs5030737), 54 (allele B, rs1800450) and 57 (allele C, rs1800451) are earlier described [15]–[17]. All the primers were purchased from SIGMA-ALDRICH, Finland.

### Statistical analyses

The difference between groups was analyzed and evaluated with GraphPad Prism 4, using the Chi-square test and Fisher's exact test. Two tailed *P*-value<0.05 was considered as significant. Odds ratios and 95% confidential intervals were also calculated.

## Results

### Bacterial colonizations

**Figure 1:**
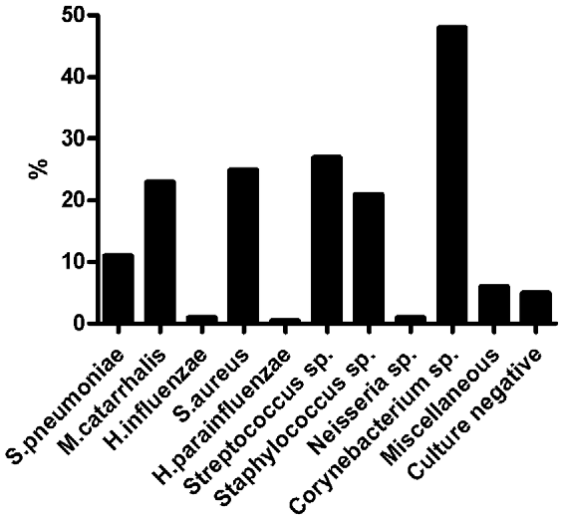
Colonizations with different bacterial species in 489 Finnish healthy infants aged at 3 months old.

Of the 489 subjects, 290 (59%) were culture positive for at least one of the four bacterial species: 55 (11%) for *S. pneumoniae*, 114 (23%) for *M. catarrhalis*, 122 (25%) for *S. aureus* and 5 (1%) for *H. influenzae* (Figure 1). Only 24 (5%) subjects were found to be culture negative. The prevalence of other bacterial genera or species is shown in Figure 1. A total of 128 (26%) subjects were positive for at least two bacterial species or genera. Of the 55 subjects who were culture positive for *S. pneumoniae*, 19 (35%) were positive for *M. catarrhalis* and 8 (15%) were positive for *S. aureus*. Four subjects were culture positive for three bacterial species: *S. pneumoniae*, *M. catarrhalis* and *S. aureus*. No significant difference was found in colonization rates of *S. pneumoniae*, *M. catarrhalis*, *H. influenzae*, and *S. aureus*, when seasonal variation was examined.

### Gene polymorphisms with bacterial colonizations

For MBL genotypes, 279 (68%) subjects were A/A homozygotes (referred as wild type), 124 (30%) were A/O heterozygotes (referred as variant type), and nine (2%) were homozygotes (referred as variant type) (Table 1). Of the 124 subjects with A/O heterozygotes, 86 were with A/B, 37 were with A/D, and one was with A/C. Of the nine subjects with O/O homozygotes, five were with B/B, two were with C/B, one was with B/D, and one was with D/D. For TLR2 genotypes, 392 (95%) subjects were C/C homozygotes (referred as wild type) and 20 (5%) subjects were C/T heterozygotes (referred as variant type), (Table 2). No T/T homozygotes of TLR2 were found. For TLR4 genotypes, 340 (83%) subjects were A/A homozygotes (referred as wild type), 69 (17%) were A/G heterozygotes (referred as variant type) and three (0.7%) were G/G homozygotes (referred as variant type) (Table 3).

**Table 1:** MBL genotypes and bacterial colonization rate (%) in 412 subjects.

	No. (%) in subjects					
	A/A (n = 279)	A/O (n = 124)	O/O (n = 9)	*Odds*-ratio	95% CI	P*
*S. pneumoniae*	27 (9.7)	24 (19.4)	1 (11.1)	2.16	1.20–3.89	0.011
*S. aureus*	55 (19.7)	40 (32.3)	2 (22.2)	1.75	1.09–2.81	0.024
*Staphylococcus sp.*	52 (18.6)	39 (31.5)	0 (0)	1.81	1.12–2.93	0.016
*M. catarrhalis*	62 (22.2)	38 (30.6)	0 (0)	1.40	0.87–2.24	0.177
*H. influenzae*	4 (1,4)	1 (0,8)	0 (0)	0.52	0.06–4.71	1.0
*Streptocoocus sp.*	82 (29.4)	34 (27.4)	0 (0)	0.83	0.52–1.32	0.482
*Corynebacterium sp.*	145 (52.0)	52 (41.9)	2 (22.2)	0.63	0.42–0.96	0.035
Culture negative	8 (2.9)	3 (2.4)	6 (66.7)	2.46	0.93–6.53	0.108

P* = A/O and O/O combined together when calculating the *P*-value.

**Table 2:** TLR2 genotype and bacterial colonization rate in 412 subjects.

	No. (%) in subjects				
	C/C (n = 392)	C/T (n = 20)	*Odds*-ratio	95% CI	*P*
*S. aureus*	86 (22.9)	9 (45.0)	2.91	1.17–7.26	0.027
*S. pneumoniae*	48 (12.2)	4 (20.0)	1.79	0.57–5.58	0.30
*M. catarrhalis*	94 (24.0)	6 (30.0)	1.36	0.51–3.64	0.593
*H. influenzae*	5 (1,2)	0 (0)	1.72	0.09–32.2	1.0
*Streptocoocus sp.*	113(28.8)	3 (15.0)	0.44	0.13–1.52	0.213
*Staphylococcus sp.*	85 (21.7)	6 (30.0)	1.55	0.58–4.15	0.408
*Corynebacterium sp.*	188 (48.0)	11 (55.0)	1.33	0.54–3.27	0.648
Culture negative	17 (4.3)	0 (0)	0.52	0.03–9.02	1.0

**Table 3:** TLR4 genotype and bacterial colonization rate in 412 subjects.

	No. (%) in subjects				
	A/A (n = 340)	A/G (n = 72)	*Odds-*ratio	95% CI	*P*
*M. catarrhalis*	72 (21.2)	28 (39.9)	2.37	1.38–4.07	0.002
*S. pneumoniae*	42 (12.4)	10 (13.9)	1.14	0.54–2.40	0.698
*S. aureus*	79 (23.2)	16 (22.2)	0.94	0.51–1.74	1.0
*H. influenzae*	4 (1,2)	1 (1,4)	1.18	0.13–10.8	1.0
*Streptocoocus sp.*	97 (28.5)	19 (26.4)	0.90	0.51–1.60	0.774
*Staphylococcus sp.*	74 (21.8.)	17 (23.6)	1.11	0.61–2.03	0.755
*Corynebacterium sp.*	165 (48.5)	34 (47.2)	0.95	0.57–1.58	0.897
Culture negative	15 (4.4)	2 (2.8)	0.62	0.14–2.77	0.748

Colonization rates of *S. pneumoniae*, *S. aureus* and *Staphylococcus sp*, were significantly higher in subjects with variant types of MBL than those with wild type (all P values<.05) (Table 1). Colonization rate of *S. aureus* was significantly higher in subjects with variant type of TLR2 than those with wild type (P = .027, Table 2). Colonization rate of *M. catarrhalis* was significantly higher in subjects with variant types of TLR4 than those with wild type (P = .002, Table 3).

## Discussion

Bacterial colonization is one of the key factors in the process of respiratory infections. Interplay between colonizing bacteria and host is closely related with the development and outcome of respiratory infections, and early nasopharyngeal colonization with pathogenic bacteria has been associated also with later development of asthma [18]. *S. pneumoniae*, *H. influenzae* and *M. catarrhalis* are highly important respiratory pathogens in both severe and mild infections, and cause the majority of cases with community-acquired pneumonia and acute otitis media. Pneumonia is responsible for 1.6 million deaths per year in children younger than 5 years of age worldwide [19]. Acute otitis media is the most common cause for antibiotic treatment in children [20]. However, little is known about why some children are colonized with these pathogenic bacteria early in life and suffer respiratory infections more often than others. This present study is part of a prospective cohort study carried out in Finland in which a large number of healthy children are included. This study provided us a unique opportunity to investigate nasopharyngeal bacterial colonization and their association with gene polymorphisms of key proteins in innate immunity.

We found that healthy infants who carry variant types of MBL have more than 2-fold increased risk to be colonized by *S. pneumonia*. Furthermore, these infants are also more often colonized by other Gram-positive bacteria such as *S. aureus* and *Staphylococcal spp*. MBL is able to bind various microorganisms including Gram-positive bacteria and plays an important role in clearance of colonizing pathogenic bacteria via phagocytosis [11], [21]. MBL can bind to carbohydrate–based pathogen-associated molecular patterns (PAMPs) on microorganism and activates complement via the MBL-associated serine proteases (MASPs) [22]–[23]. Most pneumococcal and staphylococcal strains have two types of cell wall-associated polysaccharide antigens, the capsule and teichoic acids. It is known that the capsule covers the bacterial surface during infections. MBL has a C-type carbohydrate recognition domain and its specificity has been determined to be direct against carbohydrates having equatorial '3 and '4 hydroxyl groups [24]. It has been shown that polymorphisms in the MBL structural gene can cause reduced concentrations of serum MBL but also conformational change of the protein. Our finding indicated that infants with variant types of MBL have an impaired clearance of *S. pneumonia* as well as *S. aureus*, two common bacteria colonizing nasopharynx of young infants.

In this study, we also found that young infants with variant type of TLR2 have nearly 3-fold increased risk to be colonized by *S. aureus*. However, this difference was not observed in other bacteria studied. TLR2 can recognize many ligands, most notably PGN which is a major component of the cell wall of Gram-positive bacteria [11]. It remains to be shown whether there is difference in TLR2 recognition sites of PGN between *S. aureus* and other Gram-positive bacteria such as *S. pneumonia*. The other explanation may be due to the low frequency of TLR2 Arg753Gln polymorphism observed in Finnish population. Similar frequency of the gene polymorphism was also reported in other Caucasian populations [25].

*M. catarrhalis* is a Gram-negative bacterium. Lipopolysaccharide is a major component of the outer membrane of Gram-negative bacteria, and it acts as endotoxins and elicits host immune responses. TLR4 responds strongly to LPS but this response is impaired in individuals with Asp299Gly allele. In this study, we demonstrated that infants with variant type of TLR4 are more often colonized by *M. catarrhalis*, suggesting that TLR4 plays an important role in defence against this Gram-negative bacterium.

We described nasopharyngeal bacterial colonization rates in healthy infants at 3 months of age. We found that 11% of Finnish infants were culture positive for *S. pnemoniae*. Interestingly, this percentage observed was almost the same as that found in another study carried out in Finland 10 years ago [26]. In Finland, the nationwide pneumococcal vaccination was only introduced in autumn 2010. Similar colonization rates of *S. pnemoniae* in infants were also reported in other studies [5], [27].

Soon after birth, newborn babies are colonized by *S. aureus* and its colonization rate reaches peak within a few months and start to decrease with time. Regew-Yochay, et al examined the prevalence of carriage of *S. aureus* and *S. pneumoniae* in young children in Israel and found that the carriage was 29.8% and 8.8%, at three months of age, respectively [28]. The dual carriage was only 4.3%. In this present study, the prevalence of *S. aureus* and *S. pneumoniae* was 25% and 11%, respectively and the dual carriage was 2%. It is known that there are competitive interactions between *S. pneumoniae* and *S. aureus* [29] which could partially explain the difference in the bacterial prevalence between these two pathogens.

A recent study conducted in the healthy Dutch children showed that *M. catarrhalis* colonization increased from 11.8% at age 1.5 months to 29.9% at 6 months [30]. Our finding on *M. catarrhalis* colonization in Finnish healthy infants was 23% at 3 months. Of the 114 infants who were colonized by *M. catarrhalis*, 17% were also colonized by *S. pneumoniae*. Further studies are needed to illustrate why co-colonization by *M. catarrhalis* and *S. pneumoniae* are common.

In this present study, the prevalence of colonization of *H. influenzae* was 1% in Finnish infants. This low prevalence might be due to that the study subjects are 3 months old, and the carriage of *H. influenzae* is low in young infants [31]. Moreover, the colonization rates reported can vary in infants among different countries [5], [31]. Another explanation might be the low volume (10 µl) of bacterial suspension used for culture. However, this culture volume used did not affect other bacterial findings of this study.

Many SNPs have been discovered in MBL, TLR2 and TLR4. In this study we concentrated on three polymorphisms in MBL, one in TLR2 and one in TLR4. The functions of these SNPs are well described. TLR2 polymorphism Arg753Gln frequency was found to be 5% in Finnish population. A similar frequency for this polymorphism was reported in German population [32]. The frequency of TLR4 polymorphism Asp299Gly observed in this study was 18%, which was almost identical to previous studies carried out in Finland [33]. In this study we did not examine polymorphisms of TLR4 Thr399Ile and promoter region of MBL gene. It is known that polymorphisms of TLR4 Asp299Gly and Thr399Ile are generally co-segregating in Caucasian population [34]. The frequency of SNPs in MBL was 32%; heterozygote variant 30%, homozygote variant 2%. There are other mutations in MBL that have been identified in promoter region of MBL but we concentrated on the ones located in exon1 which consist approximately 80% of all SNPs identified in MBL.

In conclusion, healthy infants who carry variant types of MBL, TLR2 or TLR4 have an increased risk to be colonized by *S. pneumonia*, *S. aureus* or *M. catarrhalis*, respectively. The finding supports a role for these genetic variations in susceptibility of children to respiratory infections. To our knowledge, this is the first study to show there is an association between nasopharyngeal bacterial colonization and genetic variation of MBL, TLR2 and TLR4 in young infants.
